# From transit hub to major supplier of illicit cigarettes to Argentina and Brazil: the changing role of domestic production and transnational tobacco companies in Paraguay between 1960 and 2003

**DOI:** 10.1186/s12992-018-0413-2

**Published:** 2018-11-19

**Authors:** Roberto Magno Iglesias, Benoît Gomis, Natalia Carrillo Botero, Philip Shepherd, Kelley Lee

**Affiliations:** 1 Center of Studies of Integration and Development, Rua Jardim Botanico 635/906, Rio de Janeiro, RJ 22470-050 Brazil; 2 Faculty of Health Sciences, Simon Fraser University, Blusson Hall, 8888 University Drive, Burnaby, BC V5A 1S6 Canada; 3 Department of Management & International Business, College of Business, Florida International University, Modesto A. Maidique Campus, 11200 S.W. 8th St, MANGO 425, Miami, FL 33199 USA

**Keywords:** Paraguay, Latin America, illicit tobacco trade, transnational tobacco companies, tobacco industry, Tobacco control

## Abstract

**Background:**

Paraguay has reportedly been a major transit hub for illicit tobacco products since the 1960s, initially to supply markets in Argentina and Brazil and, more recently, other regional markets and beyond. However, to date there has been no systematic analysis, notably independent of the tobacco industry, of this trade including the roles of domestic production and transnational tobacco companies (TTCs). This article fills that gap by detailing the history of Paraguay’s illicit cigarette trade to Brazil and Argentina of TTC products and Paraguayan production between 1960 and 2003. The effective control of illicit cigarette flows, under Article 15 of the World Health Organization (WHO) Framework Convention on Tobacco Control (FCTC) and the Protocol to Eliminate the Illicit Trade in Tobacco Products, requires fuller understanding of the changing nature of the illicit trade.

**Methods:**

We systematically searched internal industry documents to understand the activities and strategies of leading TTCs in Paraguay and subregion over time. We also mapped illicit trade volume and patterns using US government and UN data on the cigarette trade involving Paraguay. We then estimated Paraguay’s cigarette production from 1989 to 2003 using tobacco leaf flows from the United Nations Commodity Trade Statistics Database (UN Comtrade).

**Results:**

We identify four phases in the illicit tobacco trade involving Paraguay: 1) Paraguay as a transit hub to smuggle BAT and PMI cigarettes from the U.S. into Argentina and Brazil (from the 1960s to the mid-1970s); 2) BAT and PMI competing in north-east Argentina (1989–1994); 3) BAT and PMI competing in southern and southern-east Brazil (mid to late 1990s); and 4) the growth in the illicit trade of Paraguayan manufactured cigarettes (from the mid- 1990s onwards). These phases suggest the illicit trade was seeded by TTCs, and that the system of supply and demand on lower priced brands they developed in the 1990s created a business opportunity for manufacturing in Paraguay. Brazil’s efforts to fight this trade, with a 150% tax on exports to Latin American countries in 1999, further prompted supply of the illicit trade to shift from TTCs to Paraguayan manufacturers.

**Conclusion:**

This paper extends evidence of the longstanding complicity of TTCs in the illicit trade to this region and the consequent growth of Paraguayan production in the 1990s. Our findings confirm the need to better understand the factors influencing how the illicit tobacco trade has changed over time, in specific regional contexts, and amid tobacco industry globalization. In Paraguay, the changing roles of TTC and domestic production have been central to shifting patterns of illicit supply and distribution since the 1960s. Important questions are raised, in turn, about TTCs efforts to participate as legitimate partners in global efforts to combat the problem, including a leading role in data gathering and analysis.

## Resumen

**Introducción:**

según informes, Paraguay ha sido un importante país de tránsito para el comercio ilícito de productos de tabaco desde 1960, inicialmente para abastecer los mercados de Argentina y Brasil y, más recientemente, otros mercados regionales y suprarregionales. Sin embargo, hasta la fecha, no ha habido un análisis de este comercio que sea sistemático e independiente de la industria tabacalera, y que incluya los roles de la producción nacional y de las empresas tabacaleras transnacionales (TTCs por sus siglas en inglés). Este artículo llena este vacío al detallar la historia del comercio ilícito de cigarrillos - productos de las TTCs - de Paraguay con destino a Brasil y Argentina, así como la producción paraguaya entre 1960 y 2003. El control efectivo de los flujos ilícitos de cigarrillos, según el Artículo 15 del Convenio Marco de la OMS para el Control del Tabaco (CMCT OMS) y el Protocolo para la eliminación del comercio ilícito de productos de tabaco, requiere una mayor comprensión de la naturaleza cambiante del comercio ilícito.

**Métodos:**

llevamos a cabo una búsqueda sistemática en los documentos internos de la industria para entender las actividades y estrategias de las TTCs en Paraguay a través del tiempo. También mapeamos el volumen y los patrones de comercio internacional utilizando datos del gobierno de los EE. UU. y de las Naciones Unidas sobre el comercio de cigarrillos involucrando a Paraguay. Luego calculamos la producción de cigarrillos de Paraguay entre 1989 y 2003 utilizando datos sobre el comercio de hojas de tabaco provenientes de la base de datos de estadísticas de comercio internacional COMTRADE.

**Resultados:**

identificamos cuatro fases en el comercio ilícito de tabaco que involucra a Paraguay: 1) Paraguay como centro de tránsito para contrabandear cigarrillos de BAT y PMI de los EE. UU. a Argentina y Brasil (desde la década de 1960 hasta mediados de la década de 1970); 2) BAT y PMI compitiendo en el noreste de Argentina (1989-1994); 3) BAT y PMI compitiendo en el sur y sureste de Brasil (mediados a fines de la década de 1990); y 4) el crecimiento del comercio ilícito de cigarrillos fabricados en Paraguay (desde mediados de la década de 1990 hasta 1998). Estas fases sugieren que el comercio ilícito fue facilitado por las TTCs, y que el sistema de oferta y demanda de marcas de bajo precio que desarrollaron en la década de 1990 creó una oportunidad de comercio para la fabricación en Paraguay. Los esfuerzos de Brasil para combatir este comercio con un impuesto del 150% sobre las exportaciones a los países latinoamericanos en 1999, impulsaron aún más el paso de la oferta del comercio ilícito de las manos de las TTCs a los fabricantes paraguayos.

**Conclusión:**

este artículo aporta evidencia adicional sobre la complicidad de larga data de las TTCs en el comercio ilícito dentro la región y del crecimiento de la producción paraguaya en los años noventa. Nuestros hallazgos confirman la necesidad de una mejor comprensión de los factores que han influido en los cambios del comercio ilícito de productos de tabaco a través del tiempo, dentro de contextos regionales específicos y en medio de la globalización de la industria tabacalera. En Paraguay, los roles cambiantes de las TTCs y de la producción nacional han sido fundamentales para modificar los patrones de oferta y distribución desde los años sesenta. A su vez, se plantean preguntas importantes sobre los esfuerzos de la industria para participar como socios legítimos en los esfuerzos globales para combatir el problema, incluido un papel principal en la recopilación y el análisis de datos.

## Background

The illicit tobacco trade^1^ remains a major problem worldwide despite the successful legal prosecution of transnational tobacco companies (TTCs) in several jurisdictions [1–3], creation of regional initiatives to tackle the issue [4–8], the ratification of the World Health Organization (WHO) Framework Convention on Tobacco Control (FCTC) (article 15), and the entry into force of the Protocol to Eliminate Illicit Trade in Tobacco Products. According to Joossens and Raw [9], one of nine (11.6%) cigarettes smoked worldwide is illicit. This trade undermines tobacco control efforts including higher taxes by increasing the affordability of tobacco products [10–12]. Increasing affordability leads to more consumption of cigarettes, especially among the poor and youth. This, in turn, increases the incidence of tobacco-related disease and death. One conservative estimate is that eliminating the global illicit tobacco trade would save approximately 164,000 lives annually from 2030 onwards, with 32,000 lives saved in high-income countries and 132,000 in low- and middle-income countries [12]. In relation to tax revenues, governments lose an estimated US$40.5 billion per year due to cigarette smuggling [12].

Cigarette smuggling has previously been identified as playing an important role in the arrival of TTCs in Latin America, facilitating the takeover of domestic companies between the 1960s and 1980s. Shepherd [13, 14] identified TTCs as complicit in smuggling their own products into previously closed markets in Latin America and the Caribbean (LAC). Millions of pages of internal industry documents, released from the mid-1990s by whistle-blowers and through US litigation, provided further evidence of these strategies in LAC [15–19] and other regions [20–24]. Despite these revelations, and litigation against TTCs [25–27], Latin America remains “plagued by illicit tobacco” [28]. The region is described as having one of the highest levels of illicit tobacco product  penetration in the world [29, 30].

Paraguay has reportedly been a major transit hub for illicit tobacco products in the region since the 1960s. Today, Paraguay is reported to be the largest supplier of illicit cigarettes in the region. A 2002 study of the business strategies of Philip Morris International (PMI)^2^ and British American Tobacco (BAT) in the LAC region, commissioned by the Pan American Health Organization (PAHO), reported “the industry’s knowledge of and participation in the distribution of cigarettes through illegal channels” [18]. A study of the economics of tobacco control in Brazil, using official trade data on cigarettes and tobacco leaf between Brazil and other Mercosur countries from the 1990s to the early 2000s, identified unusually high levels of cigarettes stocks in Paraguay beginning in the 1990s [31]. A 2009 study by Ramos [32], which analyses the illicit cigarette trade in Mercosur countries^3^ during the 2000s, provides the most comprehensive data to date. The study describes a production boom in Paraguay, along with estimated volumes of illicit trade in local brands, using data on official trade and seizures by law enforcement, public documents, and media sources.

Investigative journalists have provided further evidence suggesting Paraguayan involvement in the illicit tobacco trade. In its *Tobacco Underground* series [33], the International Consortium of Investigative Journalists (ICIJ) reported on Paraguay’s emergence as “a top producer of contraband tobacco” [34, 35]. The election of Tabesa’s founder Horacio Cartes as President of Paraguay in 2013 prompted allegations in Brazilian newspapers *Gazeta do Povo* [36] and *Folha de São Paulo* [37]; Colombian newspapers *El Tiempo* [38–42] and *El Espectador* [43]; and *Insight Crime* [44–46]. The articles reported on Paraguay’s increasing cigarette production, smuggling routes, and relationship with organized crime in the region based on official data from the Paraguayan and Brazilian governments, and interviews conducted in the tri-border (Paraguay-Brazil-Argentina) area, including with BAT and PMI representatives.

To date, there has been limited scholarly analysis of Paraguay and the illicit tobacco trade over time. Existing studies focus on the illicit trade and tobacco control in neighbouring countries or the region as a whole. For example, using estimates of per capita cigarette consumption in Brazil and Paraguay between the early 1970s and late 1990s, and the illicit market in Brazil, Shafey et al. report that a large proportion of cigarettes legally exported from Brazil to Paraguay appeared to return to Brazil illegally [47]. Similarly, using data on prices, sales and trade, Sáenz de Miera-Juárez and Iglesias argue that Brazilian companies became complicit in the illicit market during the 1990s in response to declining local demand for higher-priced taxed legal products. They write that Brazilian companies increased legal (tax exempt) exports to Paraguay for the purpose of increasing the volume of illegal re-exports back to Brazil tax free. The authors conclude that Brazilian measures to curtail re-exports in the late 1990s came too late. By the early 2000s, Paraguayan factories were able to supply their own manufactured cigarettes to the illicit export market to Brazil [48].

While available sources provide important evidence of Paraguay’s role as a major source of illicit tobacco products to Brazil and Argentina, there is no detailed study to date of the history of the tobacco industry in Paraguay, and its development into a regional producer for “cheap whites” since the mid-1990s. This paper aims to fill this gap by: a) analysing the complicity of TTCs in seeding this trade from the 1960s; b) explaining why and how Paraguayan manufacturers became involved in the trade from the 1990s; and c) describing the rapid growth in Paraguay an manufacturing and export from the mid-1990s. In this way, this article contributes better understanding of the role of Paraguay in the changing nature of the illicit tobacco trade. Joossens and Raw [9] argue that, while the main form of illicit trade during the 1980s and 1990s was large-scale smuggling of TTC brands, other modes have since emerged in the form of “illegal manufacturing, including counterfeiting and the emergence of new cigarette brands, produced in a rather open manner at well-known locations, which are only or mainly intended for the illegal market of another country” [9]. Understanding more fully the economic factors that have shaped current nature of illicit trade, in turn, informs effective implementation of Article 15 of the FCTC and the Protocol to Eliminate the Illicit Trade in Tobacco Products.

## Methods

We began by reviewing the existing English, Spanish, and Portuguese-language literature to identify what is known about Paraguay and the illicit tobacco trade. We searched the mass media using Lexis-Nexis and Google; the scholarly literature using JSTOR, Google Scholar, and PubMed; and the grey literature using Google, key informants, and snowballing technique. These sources were used to triangulate findings from other data sources to enhance validity and reliability.

To understand the activities and strategies of TTCs in Paraguay and the region, we systematically searched the Truth Tobacco Industry Documents (TTID) collection using the keywords ‘Paraguay’ and ‘Tabesa’, combined with ‘smuggling’, ‘contraband’, and known euphemisms for illicit trade, namely ‘DNP’ (duty not paid), ‘transit’, and ‘General Trade’ (GT) [21, 49]. The snowball technique was used to generate additional keywords from reviewed documents, largely names of individuals, companies, projects and brands. Around 3000 documents were reviewed, of which 135 were found to be relevant to the themes of this analysis. The documents are from BAT, due to their legal provenance and relatively candid nature compared to other TTC internal documents available [50], thus offering particular insights on the company’s own activities, along with its subsidiaries and partners during the 1990s. The focus on BAT documents was also justified by its dominant position in the region’s cigarette markets. At the same time, the documents reviewed describe PMI strategies and activities albeit from BAT’s perspective. In available BAT documents, for example, blame for the first illegal tobacco movements in the region is placed on PMI, while BAT portrays itself as obliged to react to PMI’s actions. We do not have independent evidence to confirm which company initiated the illicit tobacco trade in northeast Argentina and Brazil. Instead, we document how both TTCs competitively participated in the illicit trade, as reflected in trade volumes over time. First, we discuss the reasons why BAT was  interested in entering the illicit market independently of PMI’s actions. These explanations go beyond the existing literature, such as PAHO (2002) which concludes that PMI initiated, and BAT reacted without noting that the only available documents were from BAT. Second, we use publicly available trade data to show trade movements in a more comprehensive way than previously documented.

For the relevant documents, we followed Forster’s hermeneutic approach to analysing company documents which emphasises reliability, contextualization and validation [51, 52]. Forster incorporates identification of meaning and categorization themes; interpretation and contextualization within geographical, temporal and corporate culture contexts; triangulating with other resources to broaden understanding of issues described; and validation of findings. Using this approach, we organized documents by date, combined with information from scholarly and secondary sources, and then iteratively reviewed to build a narrative.

In addition, we triangulated findings from the TTID, by estimating volumes of illicit trade by compiling official data on cigarette exports and imports between Paraguay and its trading partners using the United Nations Commodity Trade Stratistics Database (UN Comtrade). For the 1960s and 1970s, when UN Comtrade data is unavailable, we used data from the US Department of Agriculture (USDA) Foreign Agricultural Circular and other US government agricultural services sources. Given the lack of official data on cigarette production and grey literature on activities of Paraguayan cigarette firms, we estimated Paraguayan cigarette production from 1989 to 2003, as the local industry began to emerge. This gave us an assessment of the growth and aggregate dimension of Paraguayan’s firm activities, given that TTCs had no production facilities during the 1990s. For this purpose, we collected data on tobacco leaf exports and imports to Paraguay from UN Comtrade (see Appendix for further explanation of our methodology).

## Results

We identify four phases in the illicit tobacco trade involving Paraguay: 1) Paraguay as a transit hub for smuggling BAT and PMI cigarettes from the U.S. into Argentina and Brazil (from the 1960s to the mid-1970s); 2) BAT and PMI competing in the north-east of Argentina (1989–1994); 3) BAT and PMI competing in southern and south-eastern Brazil (mid to late 1990s); and 4) growth in the illicit trade of Paraguayan manufactured cigarettes (from the mid-1990s). We describe each of these phases to understand why and how the illicit trade in the region has changed over time, including the role of TTC complicity in the 1990s, which created a business opportunity for Paraguayan tobacco companies. The main Paraguayan company emerging from this process, of growing production and illicit sales, was Tabacalera del Este, known as Tabesa. A detailed analysis of the expansion of its manufacturing capacity since the late 1990s, to become a leading regional, and increasingly global, source of illicit tobacco products is presented in an accompanying paper (Gomis B, Lee K, Carillo Botero N, Shepherd P, Iglesias R: “We think globally”: the rise of Paraguay’s Tabacalera del Este as a threat to global tobacco control, forthcoming).

### A strategic location for TTCs to “re-export to other countries” during the 1960s and 1970s

Documents suggest Paraguay became a hub for the illicit tobacco trade during the 1960s when TTCs began to use the country to introduce US brands into the sub region. While generating immediate revenues was desired, the ultimate purpose of this trade was to facilitate the takeover of local firms in major markets in the LAC region with potential for substantial growth notably in Argentina, Brazil and Venezuela [14]. Analysing this strategy, Shepherd [14] found that, in addition to conventional methods for growing foreign markets (i.e. legal exports, licensing, joint ventures, acquisitions), TTCs used contraband channels to “soften up” local markets [53]. By channeling their brands into these markets via the illicit trade, TTCs effectively competed with and eventually weakened local companies [13, 14].

The initial supply route for US-manufactured cigarettes, during the 1960s, was to be first legally exported to small, intermediary “free trade zones” or countries like Paraguay, the Netherlands Antilles, and Panama. Available data suggests such exports would be five to ten times the level of potential consumption locally [14]. The cigarettes were then illegally re-exported in large quantities to their final destination in nearby protected markets –Argentina and Brazil in the case of Paraguay [14].

Available data suggests that, before the early 1960s, Paraguay was not a major market for US cigarette exports. Paraguay was not among the fifty countries listed as export markets in 1957–58 [54]. From the early 1960s, exports to Paraguay began to grow rapidly, climbing to almost 2 billion sticks annually by 1968 and 1969 [55]. Paraguay had become the leading importer of US cigarettes in Latin America [56], and among the top four worldwide between 1965 and 1968 [57, 58].

Table 1 describes this growth alongside an estimate of the amounts of illicit trade into Argentina from the 1960s to the mid-1970s. It highlights two important points. First, levels of US exports far outpaced the consumption capacity of Paraguayans. The population of Paraguay aged 15–64 years was around 1 million in 1965 and 1.25 million in 1970 [59]. US exports to Paraguay peaked at 1.77 billion sticks in 1968, representing per capita consumption of between 1416 and 1770 sticks. This level of consumption would not have been impossible, with US adult consumption for instance at around 2800 sticks per capita, and around 1260 sticks in 1966 and 1280 sticks in 1967 in Argentina [60]. However, this was unlikely for Paraguay, which had at the time a much lower GDP per capita than Argentina. Instead, the intended end market for American exports to Paraguay is suggested in a 1962 Brown&Williamson document which describes “American cigarettes…imported into Paraguay on an intransit basis, for re-export to other markets” [61].

**Table 1:** The rise of Paraguay as an illicit transit hub from the US to Argentina in the 1960s

Year	Cigarette production in Paraguay (million sticks)	US legal cigarette exports to Paraguay (million sticks)	Estimated cigarette contraband in Argentina (million sticks)^a^
1951–60 avg	494	*No Data*	520
1961	435	169	3200
1962	480	49	200
1963	422	204	2000
1964	520	684	2400
1965	598	967	1600
1966	574	1270	2400
1967	418	1423	3000
1968	366	1770	3800
1969	346	742	4000
1970	366	574	3600
1971	400	560	3200
1972	640	216	800
1973	620	549	1800
1974	630	865	1000
1975	640	783	*No data*
1976	650	804	*No data*
1977	*No data*	684	*No data*
1978	*No data*	856	*No data*

Sources: Cigarette Production in Paraguay: USDA, Foreign Agriculture Service, World Tobacco Analysis: Consumer Marketing, February, 1958

USDA, Foreign Agricultural Circular-Tobacco, various years, 1958-77

US legal cigarette exports to Paraguay: USDA, Foreign Agricultural Circular-Tobacco, various years, 1960-63

USDA, Foreign Agricultural Circular-Tobacco, various years, 1963-1978

USDA, Economic Research Service, Tobacco Situation, various years 1967-1990

^a^Estudio Sur, Estudio de la demanda de tabaco nacional , Buenos Aires, 1975, Cuadro A-17: 24 and Anexo C, Cuadro C-4: 81

Second, the dramatic rise in US exports to Paraguay coincided with the “denationalization” and gradual takeover of local tobacco companies in Argentina by TTCs [14]. From 1964 to 1971, as TTCs, except BAT, steadily bought up Argentine companies, Paraguay was listed by the USDA as among the top destinations for US exports. Once denationalization was completed in the early 1970s – with an industry of 5–7 major tobacco companies progressively reduced to two main ones, BAT and PMI [14], Paraguay disappeared from the list [62]. Two detailed studies corroborate the trends illustrated in Table 1. They point to a significant influx of contraband cigarettes into Argentina before PMI’s’ entry in the country around 1966, a brief period of continued smuggling thereafter to establish their international brands, and then a marked reduction in the 1970s [63]. In short, the data suggests that TTC exports were closely linked to the goal of taking over the local industry in Argentina.

Notably, these fluctuations in the illicit trade during this period did not seem to have an impact on production in Paraguay. As Table 1 (Column 2) shows, until the end of the 1970s, Paraguay’s domestic output of cigarettes was small and most likely consumed locally. Paraguay produced the lowest percentage of filter cigarettes (1–3%) out of 73 countries from 1965 to 1971 [64]. This modest production of mostly unfiltered cigarettes suggests Paraguay’s local industry unlikely played a major role in the illicit trade during this period [3].

Evidence suggests there were no major smuggling activities using Paraguay as a transit hub between the mid-1970s and end of the eighties. In those years, PMI was established in Brazil and Argentina, producing brands formerly smuggled. BAT consolidated its dominant position in both markets, in some cases acquiring new local companies (i.e. Piccardo in Argentina), and fully incorporating international brands in to the subsidiary’s portfolio to influence the taste of smokers aiming to consume “sophisticated” international brands, such as Pall Mall and Lucky Strike in Argentina.

### The battle for the north-eastern Argentinean market via Paraguay (1989–1994)

In 1989, the Argentinean economy experienced a combination of hyperinflation and output contraction, which severely depressed the purchasing power of its population [65]. This crisis pushed smokers towards cheaper cigarettes. According to BAT documents, PMI decided to develop a DNP (duty not paid, an industry euphemism for contraband market in Argentina to increase domestic supply of cheaper cigarettes. The DNP market was further expanded in 1990, “with growing exploitation by Philip Morris (with exports from Paraguay) of brands such as *Master*, *Palace* and *Mont Blanc*.... PMI share of the DNP low segment [cheaper brands] reached 87%” [66]. Meanwhile, PMI still dominated the higher-priced illicit market as well: “The DNP high segment represents 50% of total DNP volume and *Marlboro* is also the leading brand in the total DNP segment in Argentina. Practically all of this volume arrives via Paraguay” [66].

BAT documents describe how the company initially sought to counteract PMI by increasing the legal sales of low-priced *Ritz* brand cigarettes in southern Brazil, allowing their purchase by Argentinean traders [67]. By the end of 1990, however, BAT subsidiaries agreed to scale up of supplies to Argentina via Paraguay. BAT Brazilian subsidiary Souza Cruz “decided to initiate ‘exports’ of *Ritz* through the DNP route. This action was taken with the knowledge of [BAT Argentinean subsidiary] Nobleza Piccardo”[67]. By the end of 1992, Souza Cruz had “reversed the dominance of the DNP low price segment by Philip Morris, raising its share of the segment from 33% in 1990 to 68% by the end of 1992” [67].

Table 2 shows the evolution of the cigarette trade, from Brazil and the US to Paraguay, from 1989 to 1994. The table points to four key findings. First, imports reported by Paraguay from Brazil and the US account for almost all reported cigarette imports (87–98%) during this period. Second, there are major discrepancies in imports reported by Paraguay and exports reported by Brazil and the US. This difference, according to Merriman (2003) and others^4^ [68, 69], suggests illicit activity. Notably, Paraguay reported less than seven metric tonnes of imports from Brazil between 1989 and 1994, while Brazil reported over 21,000 metric tonnes of exports to Paraguay. In 1989 and 1990, the US reported no cigarette exports to Paraguay, while Paraguay reported over 3300 metric tonnes of cigarette imports from the US.

**Table 2:** Illicit trade from Brazil and US via Paraguay in the early 1990s

	Cigarettes from Brazil		Cigarettes from the USA		Brazil + USA		
	Exports to PGY reported by BRA	Imports from BRA reported by PGY	Exports to PGY reported by USA	Imports from USA reported by PGY	Imports from BRA & USA reported by PGY	All imports reported by PGY	All imports reported by PGY (in billion sticks)^a^
1989	401	0	0	1566	1566	1632	1.6
1990	1365	0	0	1759	1759	1787	1.7
1991	2586	0	2478	3877	3877	3995	3.8
1992	5703	5	2196	3277	3282	3747	3.6
1993	11,118	2	2594	3488	3490	3668	3.5

Source: UN Comtrade, HS Code 240220: ‘Cigarettes; containing tobacco’

In metric tonnes

^a^Conversion from metric tonnes to cigarettes: 1 kg = 956.4 cigarettes [31]

Third, while the US remained a major cigarette exporter to Paraguay during this period, Brazil took over as Paraguay’s main supplier by 1992, exporting over four times more cigarettes to Paraguay than the US did in 1993.

Fourth, population or income growth alone cannot account for the increased flow of cigarettes to Paraguay between 1989 and 1993. The Paraguayan adult population (15 or above) grew from 1.7 to 1.85 million [70], while the country’s GDP per capita increased by 9.8%. On this basis, the expected growth of the domestic market for imported cigarettes would have been approximately 19.4%.^5^ Instead, Table 2 shows reported imports more than doubling, from 1.6 to 3.5 billion sticks [71].

Documents suggest that the subsequent rapid increase in exports, to Argentina via Paraguay by BAT Brazillian subsidiary Souza Cruz, created conflict its Argentinian subsidiary Nobleza Piccardo, warning that illicit sales of *Ritz* in Argentina would “prejudic[e] the market share and profitability of both the Group and [Nobleza Piccardo]” [18, 66]. BAT responded to these tensions by launching the Pampa Project in 1992, forming an internal group to analyse the optimal share of the illicit market between the two subsidiaries to maximize BAT profit [18]. Souza Cruz and Nobleza Piccardo agreed to “[c]onstruct and implement specific marketing actions with a view to maximizing group profit from the DNP trade [and] leverage [BAT] Group long-standing strength in the DNP region” [66]. In late 1992, the Pampa Project proposed:

1. Export *Jockey Club* [produced in Brazil] to Paraguay … for re import to the N.E of Argentina…2) Exploit the existing *Derby* franchise with the ‘slims’ version, produced in Brazil exported to Paraguay for distribution in the N.E.A…and 3) Fulfil the market demand in the DNP low segment with *Ritz* and *Belmont* … in order to achieve a market share of 75% [66].

Company analysis of the financial implications of these proposals determined that high volume and prices for *Jockey Club* and *Derby* would be needed to achieve reasonable profit. As this was deemed not feasible at the time, it was agreed in 1993 to maintain the status quo in the illicit Argentinean market [67, 72–75]. The status quo for Souza Cruz and Nobleza Piccardo was continued exports to Paraguay for illegal re-export purposes. Brazilian exports to Paraguay almost doubled in 1992–1993, reaching 11,000 metric tonnes or more than 10 billion sticks (Table 2). Documents describe Souza Cruz and Nobleza Piccardo continuing to “recycle…products through Paraguay and back into their respective markets making use of lower excise rates in Paraguay” [71, 76]. For BAT, illicit cigarettes in the region were seen as a “fact of life and almost institutionalized” [77], and the illicit

“[r]e-export of a wide range of goods has been a traditional source of business in Paraguay’s economic history….The significant difference in excise tax among neighbouring countries in the southern cone region, will continue to encourage DP imports into Paraguay to be followed by DNP re-exportation” [71].

For Paraguayan domestic manufacturers, the supply route for illicit cigarettes to Brazil and Argentina created by TTCs offered a new business opportunity. A 1993 BAT document suggets the company became aware that Paraguayan companies had also begun to supply the illicit markets into Brazil and Argentina:

Approximately 100 Mns. US International brands of US manufacture and legally imported into Paraguay cross into Argentina as contraband monthly. Philip Morris accounts for most of this business, mainly *Marlboro*…. Approximately 200 Mns. cigarettes of Brazilian manufacture per month use Paraguay as a route into Argentina (not paying Paraguayan taxes on route). Souza Cruz hold 70 p.c. [percent] of this business, mainly *Ritz*. Paraguayan local brands are also reported to enter Argentina illegally, approximately 20 Mns per month [71].

A 1994 BAT document identifies Paraguayan company La Vencedora (LVA) as part of the illicit market in Brazil holding at least 20% of the market, mainly in sales of *Pink Hollywood* [78]. Notably, the ability of Paraguayan manufacturers to compete in the illicit trade challenged a previously documented strategy used elsewhere by TTCs - to create an illicit market when needed to access a closed market; increase demand for and sales of their brands; and then close the market in response to a change in government policy, in order to exclude competitors [79]. In Argentina, BAT understood that “we must be prepared to vacate the D.N.P segment completely without leaving a vacuum which…competitors are better placed to fill than ourselves” [80]. The post-exit strategy was to continue legally supplying the region, as “customer franchise” had been developed. BAT considered itself in a position to open and close the illicit market in future if necessary [80].

However, the low-priced segment of the illicit market in Argentina was different. BAT recognized that, although “*Ritz* enjoyed significant consumer preference”, “the principal consumer purchasing motivator in the DNP low price [segment was] price” [66]. There were other lower-priced competitors in the market:

…when *Ritz* was not available the consumer switched brands, in order of preference, to falsified *Ritz* (Paraguay), P[hilip] Morris brands and finally other Paraguayan brands. This was demonstrated recently when temporary shortages of *Ritz* (*S. Cruz*) were experienced due to strikes by Brazilian Receita Federal (Customs Authorities) [66].

In short, new competitors (including Paraguayan companies LVA and Boquerón) were now recognized as producing low-priced brands, and competing with TTCs in the subregion’s illicit market.

### The fight for market share in Brazil and the “triangular scheme”

The so-called ‘triangular scheme’ in the 1990s consisted of exporting Brazilian cigarettes to Paraguay, particularly after 1993, and illegally reintroducing those products in the Brazilian market [31, 32, 48]. The Brazilian illicit cigarette market grew rapidly from approximately half a billion sticks in 1992 to 8 billion sticks in 1993 [81]. Three factors explain BAT’s decision to increase its complicity in the illicit trade, through legal exports from Brazil to Paraguay and illegal illegal re-export back to Brazil.

The first factor was related to competition between BAT and PMI. BAT’s Brazilian subsidiary, Souza Cruz, alleged that it entered into the illicit market in response to PMI’s efforts to increase market share by selling lower-priced brands through legal and illegal channels [82–84]:

[F]rom October 1993 onwards Souza Cruz is planning to carefully step-up its DNP efforts with a view to achieving a competitive share in the segment. A full brand portfolio is being launched, composed of international and local offers with competitive edge against PM brands [82].

BAT’s goal was to achieve a 40% share of the illegal market by 1998 [81]. Although Souza Cruz’s official position was that PMI made the first move into the Brazilian illicit market, the BAT subsidiary also recognised that PMI was, in part, responding to the introduction of *Derby*, produced by BAT’s Argentine subsidiary Nobleza Piccardo, into the Brazilian “transit business” [83]. PMI itself then launched *Dallas* in the first half of 1993, after increasing exports to Paraguay earlier that year, as part of a portfolio of brands for the illicit Brazilian market [82]. In response, Souza Cruz moved forward its plans to launch *Derby* in Brazil – including in the illicit market - from 1995 to 1993, and “implement[ed] several actions” to control PMI’s share of the illicit market [82, 85].

The second factor was the depressed economic climate. The 1990 recession in Brazil led to a decline in cigarette sales. On top of that, Souza Cruz and PMI increased retail prices over inflation rates in late 1991 to restore profit margins [82, 86]. Real price increases, alongside economic stagnation, reduced the purchasing power of smokers and thus legal sales. In addition, Souza Cruz reported that smaller Brazilian companies such as Sudan, Cibrasa, and Ciamerica had underreported their tax obligations, and thus were able to sell at lower prices, gaining a price advantage over Souza Cruz by the end of 1992 [87]. Souza Cruz’s market share declined from 82.2% in 1992 to 78.8% in 1993, with lost market share going to PMI and Brazilian companies [83]. Documents suggest BAT responded to these developments by increasing the supply of its brands through the illicit trade.

A third factor was increasing competition from Paraguayan cigarettes. A BAT document describes growing sales by Paraguayan companies in the tri-border region, notably Ciudad del Este, competing with each other [88], and threatening to take market share from TTCs in Brazil. Paraguayan sales were comprised of two types of products: counterfeits (e.g. Souza Cruz brands *Ritz* and *Minister* copied by Boquerón) and licensed brands intended for exclusive sale in Paraguay (e.g. BAT brands *Pink Hollywood*, *Continental* and *Advanced,* manufactured under license by LVA). Both types of brands were now entering the Brazilian market illegally, competing alongside TTCs in southern Brazil [76, 89–93]. In a letter to Keith Dunt (BAT), Flavio de Andrade (Souza Cruz) noted unsuccessful legal actions against Boqueron to stop the counterfeiting of Souza Cruz brands in Paraguay [82, 89, 94]. However, on BAT’s suggestion to withdraw *Ritz* from Ciudad del Este, de Andrade wrote that this “could create a favourable condition for Boquerón to increase [illicit] sales in the Brazilian market, without competition, threatening Souza Cruz’s domestic sales” [85].

Documents suggest BAT’s response to licensed brands produced by LVA was different. The idea of partnering, or even acquiring, parts of the company had been discussed since the mid-1970s [95–100]. BAT’s interest in investing in Paraguay was not related to a growing domestic market, but “purely defensive” [78]^6^ to block LVA’s access to the Argentinean and Brazilian markets. Internal correspondence and visit reports from the 1990s describe prolonged and difficult negotiations with LVA, hinging on two issues: the recovery of trademarks (licensed to LVA in the 1970s) and the need to protect BAT’s place in the sub-regional market (Brazil and Argentina) after the signing of Mercosur in 1991 [76, 82, 93, 101].

Souza Cruz perceived other suppliers in the increasingly crowded illicit market, not only as a competitive threat, but as an opportunity to advocate for lower excise taxes on tobacco products. Documents describe the company seeking to convince Brazilian authorities to reduce excise taxes in order to lower the price of legal brands, arguing that this would discourage the sale of cheap illegal cigarettes [82]. Sáenz de Miera-Juárez and Iglesias [48] analyse the coordinated strategy by Souza Cruz and PMI during the early 1990s. They found that, between 1991 and 1993, both Souza Cruz and PMI increased cigarette prices in real terms, and the maintained prices at a higher level than in the 1980s. In 1993, Souza Cruz and PMI then increased tax-free exports to Paraguay, which were then re-exported illegally back to Brazil. These illicit cigarettes offered a cheaper option to smokers than domestically taxed legal cigarettes. Meanwhile, the two companies lobbied the Brazilian government to lower excise taxes on cigarettes, claiming that this would reduce the illegal market [81]. In June 1999, the Brazilian government finally reduced the excise tax per pack (from approximately 40% of retail price to about 25% [4, 86]) and authorities kept rates low in real terms into the mid-2000s, in the belief that this would discourage smuggling.

The increased presence of TTCs in Brazil from the 1990s led to another shift in the sub-region’s illicit trade. Official trade data points to an increase in volume of trade, as well as trade partners (Table 3). Although Brazilian exports to Paraguay during the 1990s have been previously documented [31, 47], what has not been analysed to date is the farther reach of the triangular scheme. Brazil not only exported higher quantities than Paraguayans could consume domestically, but also exported to Uruguay on a similar scale [31]. In addition to Brazil and the US (already accounting for almost all cigarette exports to Paraguay between 1989 and 1993 as shown in Table 2), Uruguay and Argentina began exporting large quantities of cigarettes to Paraguay between 1994 and 2002 (Table 3). The four countries accounted for almost all cigarette imports by Paraguay during those eight years.

**Table 3:** The expansion of the illicit trade in Brazil through Paraguay

	Tobacco products from Brazil		Tobacco products from Argentina		Tobacco products from the U.S		Tobacco products from Uruguay		Total		Total imports reported by PGY from all trade partners worlwide	
	Exports to PGY reported by BRA	Imports from BRA reported by PGY	Exports to PGY reported by ARG	Imports from ARG reported by PGY	Exports to PGY reported by USA	Imports reported by PGY	Exports to PGY reported by URY	Imports reported by PGY	Exports to PGY reported by BRA, ARG, US, URY	Imports reported by PGY	In metric tonnes	In billion sticks^a^
1993	11,118	2	33	47	2594	3488	0	12	13,745	3549	3668	3.5
1994	13,309	3263	1218	1813	1986	3511	0	32	16,513	8619	9025	8.6
1995	17,838	16,041	1633	2439	2200	3646	163	320	21,834	22,446	22,811	21.8
1996	12,920	24,727	1342	2167	1910	2839	3636	3340	19,808	33,073	33,745	32.3
1997	19,436	25,690	1687	2625	2239	3343	3333	4289	26,695	35,947	36,923	35.3
1998	23,355	22,093	1536	2128	2292	2152	6662	8519	33,845	34,892	35,452	33.9
1999	616	4171	2554	2983	983	1055	6434	8003	10,587	16,212	16,659	15.9
2000	0	45	1905	2147	No data in volume	887	6084	6824	7989	9903	10,321	9.9
2001	0	2	0	0	No data in volume	694	5489	4865	5489	5561	6132	5.9
2002	0	0	38	22	No data in volume	155	4669	4496	4707	4673	4865	4.7

Source: UN Comtrade, HS Code 240220: ‘Cigarettes; containing tobacco’

In metric tonnes

^a^Conversion from metric tons to cigarettes =1 kg = 956.4 cigarettes [31]

Three observations can be drawn from Table 3. First, the flow of cigarettes to Paraguay grew substantially between 1993 and 1998 (146% increase in reported exports and 883% increase in reported imports). This was notably related to the increased use of Paraguay as a transit hub for illicit tobacco by Argentina and Uruguay [31]. Cigarette shipments from Argentina to Paraguay, as reported by both partners, jumped between 1993 and 1994 and then remained stable until 2000. Documents suggest Nobleza Piccardo decided to export *Derby* and *Jockey Club* brands to Paraguay in late 1993, for re-export back to Argentina, in order to challenge the dominance of PMI in the northeast Argentine market [102]. The US, already a major exporter of cigarettes to Paraguay since 1991 ([71], Table 1, Table 2), continued to supply the Paraguayan market in excessive quantities until 1998 (consumption in Paraguay at the time was around 3 billion sticks) [71].

Second, the Brazilian government introduced a 150% tax on cigarette exports to LAC countries in 1999, with the aim of discouraging the re-report of those cigarettes via the illicit trade [4, 31]. This effectively put an end to the TTCs’ triangular scheme. Between 1994 and 1998. Brazil had become Paraguay’s main source of cigarette imports, sending four to seven times the volume of Paraguayan cigarette consumption [71]. The tax had the expected effect of sharply reducing Brazilian exports to Paraguay (Table 3).

Third, Uruguay became a major exporter to Paraguay. Between 1996 and 1998, Uruguay was second only to Brazil in exports to Paraguay, with volumes almost three times Paraguayan domestic consumption. Then from 1999, following the adoption of a new export tax by the Brazilian government, Uruguay became Paraguay’s leading export source. Uruguayan company Monte Paz manufactured most of these cigarettes, with Brazil as their final destination via the illicit trade [47].

### “A vacuum which our competitors are better placed to fill than ourselves” [80]: The boom of Paraguayan cigarette production after 1994

By 1994, TTCs no longer considered Paraguayan cigarettes as minor competition, but instead as a growing threat to their market share. As explained in the previous section, cheaper brands sold by TTCs in the illicit market did not compete on the basis of brand loyalty but on price. Low-priced Paraguayan brands started competing by price with TTC brands in northeast Argentina. In the mid 1990s, growing production capacity was being developed by Paraguayan companies. Milton Cabral (Abifumo, Brazilian Tobacco Industry Association) mentions the growth of Paraguayan cigarette companies during the 1990s in a 2001 seminar organized by the Brazilian Federal Revenue Secretariat [103]. Cabral identified two periods of growth: 1994–1996 when the number of cigarette factories in Paraguay and Uruguay increased from 6 to 12; and 2000–2001 when factories increased from 17 to 29. However, there has been limited independent research [32] of this growth, largely due to the lack of available government or company data on production.

Based on new methodology (described in Appendix), this paper (Table 4) provides estimates of cigarette production in Paraguay between 1989 and 2003. This data suggests that, between 1989 and 1994, Paraguayan production remained below the domestic consumption level of 3 billion sticks [71]. This rate of production corresponds with TTCs supplies to the illicit market in Brazil and Argentina, via Paraguay as the transit hub. Between 1995 and 1998, Paraguayan production then grew exponentially, from 4 billion to over 12 billion sticks annually, three times the size of Paraguayan domestic consumption. This was also the peak period of TTC supplies to the illicit market. This pattern suggests Paraguayan companies shifted to also supplying the illicit market in competition with TTCs. Between 1999 and 2003, following Brazil’s introduction of the aforementioned export tax on cigarettes to LAC countries, and the corresponding reduction in TTC supplies, Paraguayan production grew even further, doubling to almost 27 billion sticks by 2003, about 8 times domestic consumption. This growth suggests that Paraguayan companies grew in response to an opportunity to fill a vacuum in an illicit market created and initially supplied by TTCs.

**Table 4:** The boom of cigarette production in Paraguay

Period	Years	Estimated average annual cigarette production (billion sticks)
Before 1994	1989–1992	1.20
	1993	2.70
1994–1998	1994–1995	4.03
	1996–1997	6.78
	1998	12.38
1999–2003	1999–2000	13.68
	2001–2002	25.75
	2003	26.83

Source: See Appendix for methodology

Table 5 compares volume of imported (Table 3), and domestically produced cigarettes (Table 4) in Paraguay. The proportion of Paraguayan cigarettes in the total supply oscillated between 15.2 and 43.5% between 1992 and 1998, jumping to 64.5–69.8% in 2000, and increasing to 87.9–91.8% by 2003. Given approximately 3 billion sticks annually for domestic consumption [71], it is assumed the remainder was for export. Table 5 suggests that TTCs’ concerns in the mid-1990s, that Paraguayan cigarette manufacturers would take control of the illicit market in Brazil (as described above) materialised after the export tax was introduced in in 1999.

**Table 5:** The changing role of TTCs and Paraguayan companies

Year	Total cigarette imports from all trade partners worldwide, as reported by PGY and exporters, respectively (billion sticks) (1)	Local cigarette production (billion sticks) (2)	Total cigarette supply (billion sticks) (3) = (1) + (2)	Share of Paraguayan production in total cigarette supply in Paraguay (%) (4) = (2)/(3)
1989	1.6–0.4	0.3	1.9–0.7	15.8–42.9
1990	1.7–1.4	0.8	2.5–2.2	32–36.4
1991	3.8–4.9	2.2	6–7.1	36.7–31
1992	3.6–7.6	1.5	5.1–9.1	29.4–16.5
1993	3.5–13.2	2.7	6.2–15.9	43.5–17
1994	8.6–16	3.7	12.3–19.7	30.1–18.8
1995	21.8–21.2	4.3	26.1–25.5	16.5–16.9
1996	32.3–19.4	5.8	38.1–25.1	15.2–23.1
1997	35.3–26.2	7.7	43.0–33.9	17.9–22.7
1998	33.9–33.2	12.4	46.3–45.6	26.8–27.2
1999	15.9–10.4	9.4	25.3–19.8	37.2–47.5
2000	9.9–7.8	18.0	27.9–25.8	64.5–69.8
2001	5.9–5.4	28.4	34.3–33.8	82.8–84
2002	4.7–4.5	23.1	27.8–27.6	83.1–83.7
2003	3.7–2.4	26.8	30.5–29.2	87.9–91.8

Sources:Reported cigarette imports: UN Comtrade

Estimated cigarette production: See Appendix for methodology

## Discussion

The above findings contribute new understanding of the changing nature of the illicit tobacco trade over time, in a subregion of Latin Amerca [9]. Beginning in the 1960s, documents suggest TTCs recognised Paraguay as an ideal transit hub for illegally supplying their international brands to large markets in neighbouring countries. The initial objective of TTCs, particularly PMI, was to take control of domestic cigarette companies in Argentina. During the late 1980s and 1990s, a period of regional economic downturn, TTCs used Paraguay to establish an illegal market of lower-priced brands in the subregion. This paper argues that TTCs, by creating this type of illicit trade based on their own lower-priced brands, established the conditions for Paraguayan companies to enter and flourish. Prior to the 1980s, the Paraguayan tobacco industry had limited capacity and supplied only a relatively small domestic market. Between 1990 and 1998, cigarette production in Paraguay increased from less than one billion to twelve billion sticks, with domestic companies playing the main role in increasing production. In this way, the illicit market established by TTCs encouraged local companies to transform Paraguay, from a transit hub to a major supplier of so-called “cheap whites”, defined as “cigarettes manufactured by legitimate business enterprises with a large share of the production being sold without all applicable duties paid, usually outside the jurisdiction where they are produced” [104] (Table 6).

**Table 6:** Key moments in the history of Paraguay’s tobacco industry and TTC complicity in the illicit trade

From the 1960s	Paraguay serves as a transit hub for BAT and PMI cigarette smuggling from the US to Argentina and Brazil
Late 1980s - early 1990s	BAT and PMI increase their focus on cheaper brands, paving the way for Paraguay manufacturers to capitalize on this market
1989–1994	BAT and PMI compete in the illicit market in north-east Argentina
Mid-1990s - late 1990s	BAT and PMI compete in the illicit trade in southern Brazil
1990s	While BAT and PMI increase legal exports of cigarettes from Brazil to Paraguay for them to be smuggled back to Brazil, they lobby the Brazilian government to lower the excise tax on cigarettes, claiming this would reduce the black market. Brazil eventually reduces the excise tax per pack from approximately 40% of retail price to about 25% in 1999.
1994	Creation of Tabacalera del Este (Tabesa), soon to become Paraguay’s largest tobacco company and major regional supplier of illicit cigarettes
1994–1998	Brazil becomes Paraguay’s main source of cigarette imports (4 to 7 times the volume of Paraguayan cigarette consumption), with Uruguay becoming another major supplier.
From the mid-1990s	Domestic cigarette production and illicit trade out of Paraguay begins to grow (tripled between 1995 and 1998, doubled between 1999 and 2003 to 27 bn sticks, approximately 8 times total domestic consumption)
1999	Brazil’s 150% export tax on cigarette exports to Latin American countries ends TTC scheme but shifts supply of illicit trade to Paraguay

Our findings raise several implications for combating the illicit tobacco trade and protecting public health. First, our paper adds further evidence, not only of the complicity of TTCs in the illicit tobacco trade, but their culpability in seeding this trade in this subregion and creating the conditions for other suppliers to flourish. The BAT documents reviewed in this paper suggest BAT and PMI established substantial supply and demand for illicit low-priced cigarettes which, in turn, created the economic incentives that attracted Paraguayan manufacturers to eventually compete for this lucrative market. Current efforts by TTCs to focus attention on so-called “cheap whites”, produced by these latter manufacturers now competing against them, must be understood within this context. As Ross et al. write:

They oppose them not because they are illegal, but because they represent competition. In addition, TTCs seem to use the presence of illegal Cheap Whites to divert attention from their own contribution to the illegal cigarette market [104].

It is argued here that this culpability of TTCs, in seeding the illicit tobacco trade in this Latin American sub-region, reaffirms the need to exclude the industry from measures to address the illicit tobacco trade.

Second, the findings suggest that understanding the changing nature of the illicit tobacco trade requires deeper knowledge of the links between regional and global trends. In this paper, we have described how TTCs introduced the illicit trade into a sub-region. The flourishing of this trade, including the emergence of Paraguayan manufacturers of illicit cigarettes, has created a new source of illicit supply to markets far beyond the sub-region. Media reports have identified Paraguayan companies as an increasingly important source of illicit tobacco products worldwide. In our accompanying paper (Gomis B, Lee K, Carillo Botero N, Shepherd P, Iglesias R: “We think globally”: the rise of Paraguay’s Tabacalera del Este as a threat to global tobacco control, forthcoming), we systematically analyze the rise of the largest of them, Tabesa, as a threat to global tobacco control. This interconnectedness between regional and global developments is an important feature of the contemporary illicit tobacco trade.

Third, the paper provides better understanding of the substantial size of this illicit market and hence the devastating public health consequences for this sub-region. Poor data has previously hindered the ability to measure the scale of the illicit trade. Bringing together different data sources, our findings suggest large volumes of cheap, untaxed, and largely unregulated cigarettes have been made available in Argentina and Brazil over decades. The methodology used in this paper to measure the illicit trade may be usefully applied in other regions where official data is poor. Our findings notably demonstrate the need to monitor and control key inputs in cigarette manufacturing – including tobacco leaf, cigarette paper, and acetate tow – as part of efforts to tackle the illicit trade globally. Improved data is central to disentangling the roles of TTCs and other industry actors in the illicit trade, and a precursor to effectively implementing the FCTC Protocol to Eliminate Illicit Trade in Tobacco Products.

## Conclusion

TTCs created the conditions that enabled Paraguay to shift, from transit hub from the 1960s, to a major supplier of illicit cigarettes to the region and beyond from the mid-1990s. While BAT and PMI sought to further their interests in Brazil and Argentina through this strategy, the unintended consequence was the emergence of Paraguayan manufacturers focused on filling a vacuum in the illicit supply chain. The continued growth of domestic tobacco companies in Paraguay since the mid-2000s, as described in the accompanying paper, means increasingly important competition for of TTCs in the region and beyond.

## Appendix

### Methodology for estimating cigarette production in Paraguay

Paraguay does not have public data on duty-paid cigarette sales or any other measure of cigarette production. In order to estimate production in the 1990s and early 2000s, we followed the methodology put forth by Corradini [105], and later used in Biz [106]. The main idea is to use information on production, or availability of one or several key inputs for cigarette production in a certain year, and the proportion of a specific key input used in one unit (cigarette stick) in that country, to infer total cigarette production. Corradini’s methodology can be summarized by the following equation:

$$ \mathrm{Estimated}\ \mathrm{cigarette}\ \mathrm{production}=\mathrm{production}\ \mathrm{or}\ \mathrm{availability}\ \mathrm{of}\ \mathrm{a}\ \mathrm{key}\ \mathrm{input}/\mathrm{quantity}\ \mathrm{of}\ \mathrm{the}\ \mathrm{input}\ \mathrm{to}\ \mathrm{produce}\ \mathrm{one}\ \mathrm{cigarette}\ \mathrm{stick} $$

[105]

This methodology presents some drawbacks. Notably, alternative uses of those key inputs may divert available volumes to other final uses; changes in end-of-year stocks of those key inputs may not affect the actual levels of cigarette production in a certain year; and there may be some measurement errors.

There are several key inputs to be considered with regard to cigarettes, such as tobacco leaf, filter tow, and several types of papers used in cigarette production and packaging. Tobacco leaf is the better candidate for production estimation because of its exclusive use for cigarettes and other tobacco products. In addition, Paraguay imported tobacco leaf almost exclusively for cigarette production in those years. Filter tow is another key input exclusively dedicated to cigarette production, but the reporting of trade transactions is often subject to classification mistakes. In contrast with filter tow and cigarette paper, one can assume that tobacco leaf, a traditional and easily identifiable product, was then is subject to fewer reporting errors. For these reasons, we used tobacco leaf imports as the basis of our production estimates. Paraguay produced some native or dark tobacco during this period, but these leaves were mainly exported or used to manufacture other local tobacco products (i.e., small cigars). In some cases, dark leaves could have been mixed with tobacco used for cigarette production, but likely not in those destined for the illicit markets in Brazil or Argentina, where smokers preferred cigarettes manufactured from Virginia leaf. For this reason, we excluded local dark tobacco in our calculations, and in that we differ with Ramos (2009) [32].

The relationship between the quantity of tobacco leaves and cigarette production levels also depends on leaf processing, the types of tobacco mixtures used, and the average quantity of tobacco mixture used per cigarette. In the case of Paraguay, UN Comtrade provides data for three types of tobacco leaf imports during the 1990s.

- Tobacco, unmanufactured, not stemmed nor stripped (HS Code 240110)
- Tobacco, unmanufactured, partly or wholly stemmed and stripped (HS Code 240120)
- Tobacco refuse (HS Code 240130)

We assumed that all imported tobacco has been cured. Unmanufactured tobacco, not stemmed nor stripped, should be processed and the stem should be separated from the rest of the leaf in order to be used in the tobacco mixture to fill cigarettes. Following Corradini [105], we estimated that there is an 11% weight loss due to extraction of the stem. Moreover, we assumed that stemmed tobacco and tobacco refuse were used in full for cigarette production. Of course, with a better knowledge of the techniques used in Paraguay in the 1990s, it will be possible to make more accurate assumptions on weight losses of imported tobacco leaves during the preparation of the filler of cigarette sticks.

Finally, following Corradini [105], we assumed that the average quantity of tobacco filler used in the preparation of one cigarette stick was 0.83 g. Ramos [32] used 0.65 per stick, assuming an inferior quality of Paraguayan cigarette production. However, according to some local specialists, the inferior quality was more likely due to the higher proportion of tobacco refuse used for the cigarette filler rather than to a lower amount of tobacco in an average stick. Since we did not consider the local dark tobacco production, adjusted import volumes of not stemmed tobacco (by 11%), and assumed a higher quantity of tobacco filler per stick, our estimations of cigarette stick production is lower than Ramos [32] for example.

As is the case when illicit activities are involved, there were significant discrepancies between volumes of tobacco leaf shipped to Paraguay as reported by exporters and as reported as imports by Paraguay. During the period analyzed (1989–2003), the countries mainly supplying tobacco leaf were Brazil and Argentina. In the first half of the 1990s, reported exports of tobacco leaf from Brazil were lower than reported imports by Paraguay. After 1995, when Paraguayan firms were increasingly engaged in the illicit market in Brazil, reported imports were lower than reported exports, most likely for local manufacturers sought to conceal the volume of their operations. As reported exports are more likely as be diverted to other countries or not exported at all, we decided to use reported imports by Paraguay.

Using this methodology, Table 7 describes volume of reported leaf imports adjusted for processing requirements, and the estimated cigarette production (assuming 0.83 g of tobacco filler per stick).

**Table 7:** Tobacco leaf imports reported by Paraguay, and estimated cigarette production, 1989–2003

	Imported tobacco leaf availability for cigarette production	Estimated cigarette production	Year-on-year increase
	Metric tonnes	Billion cigarettes	Billion cigarettes
1989	231	0.3	N/A
1990	689	0.8	0.55
1991	1790	2.2	1.33
1992	1265	1.5	−0.63
1993	2220	2.7	1.15
1994	3101	3.7	1.06
1995	3590	4.3	0.59
1996	4852	5.8	1.52
1997	6408	7.7	1.87
1998	10,278	12.4	4.66
1999	7802	9.4	−2.98
2000	14,909	18	8.56
2001	23,601	28.4	10.47
2002	19,138	23.1	−5.38
2003	22,272	26.8	3.78

Source: UN Comtrade. Positions 240110-240120-240130. Volumes of 240110 were adjusted by -11%, the rest of tobacco leaves entered without losses in the tobacco fill

## Abbreviations

BAT: British American Tabaco
DNP: Duty Not Paid
DP: Duty Paid
FAOSTAT: Food and Agriculture Organization Statistics Database
FCTC: Framework Convention on Tobacco Control
IDESF: Instituto de Desenvolvimento Economico e Social de Fronteiras (Institute for the Economic and Social Dvelopment of the Borders)
ITIC: International Tax and Investment Center
LAC: Latin America and the Caribbean
LVA: La Vencedora
Mercosur: Mercado Común del Sur (Southern Common Market)
NEA: Northeast Argentina
PM: Philip Morris
PMI: Philip Morris International
RJR: RJ Reynolds
Tabesa: Tabacalera del Este
TTC: Transnational tobacco company
TTID: Truth Tobacco Industry Documents
USDA: United States Department of Agriculture
WHO: World Health Organization

## Footnotes

1 The Protocol to Eliminate Illicit Trade in Tobacco Products of the World Health Organization (WHO) Framework Convention on Tobacco Control (FCTC) defines illicit trade as “any practice or conduct prohibited by law and which relates to production, shipment, receipt, possession, distribution, sale or purchase including any practice or conduct intended to facilitate such activity” (Article 1).

2 Philip Morris International (PMI) was incorporated as an operating company of Philip Morris Companies Inc. in 1987. Because this paper discusses the company’s international operations, it uses the abbreviation PMI except for quotations from industry documents which may use the abbreviation PM.

3 The Southern Common Market (Mercosur) was established in 1991 by Argentina, Brazil, Paraguay and Uruguay. Venezuela acceded in 2006 and Bolivia in 2015. http://www.mercosur.int

4 According to Merriman (2003), “One method to detect and measure such tactics of tobacco smuggling is to compare reported tobacco exports destined for a country to that country’s reported tobacco imports. Persistent discrepancies between these amounts—discrepancies that cannot be explained by other factors—provide an estimate of the amount of wholesale smuggled tobacco.” (Merriman, D. Understand, Measure, and Combat Tobacco Smuggling. *World Bank Economics of Tobacco Toolkit*, Tool 7. 2003. http://siteresources.worldbank.org/INTPH/Resources/7Smuggling.pdf).

5 The evolution of the Paraguayan market for foreign cigarettes should have roughly mirrored that of the GDP per capita and population, i.e. (1 + 9.8%)*(1 + 8.8%) or a 19.4% increase.

6 La Vencedora transfiere todas sus marcas a British American Tobacco [LVA closed down in 2002 and sold all its brands to BAT]. *ABC Color,* 8 November 2002. http://www.abc.com.py/edicion-impresa/economia/la-vencedora-transfiere-todas-sus-marcas-a-british-american-tobacco-671206.html)
